# Evaluation of the bioaccessibility of a carotenoid beadlet blend using an in vitro system mimicking the upper gastrointestinal tract

**DOI:** 10.1002/fsn3.2295

**Published:** 2021-05-04

**Authors:** Chun Hu, Dawna Salter Venzon, Katja Lange, Annet Maathuis, Susann Bellmann, Kevin Gellenbeck

**Affiliations:** ^1^ Nutrilite Health Institute Buena Park CA USA; ^2^ TIM BV Zeist The Netherlands

**Keywords:** bioaccessibility, carotenoid, gut simulation, TIM‐1

## Abstract

The release characteristics of a unique blend of carotenoid beadlets designed to increase bioavailability were tested using the dynamic gastrointestinal model TIM‐1. Individual carotenoid bioaccessibility peaks were observed over approximately 3–4 hr in the order of lutein and zeaxanthin first, followed by lycopene, and then finally α‐ and β‐carotene; when tested as a beadlet blend or when the beadlets were compressed into tablets. Bioaccessibility measurements of 7%–20% were similar to those previously reported in literature and comparable between the two formulations, beadlet blend and tablet formulations. Total recovery of carotenoids from all compartments ranged from 70% to 90% for all carotenoids, except lycopene where almost 50% was unrecoverable after digestion in the TIM system.

## Graphical abstract

The release characteristics of a unique blend of carotenoid beadlets designed for increased bioavailability were tested using the dynamic gastrointestinal model TIM‐1. Peak bioaccessibility measurements separated over approximately 3–4 hr in the order of lutein and zeaxanthin first, followed by lycopene, and then finally alpha and beta carotene; both when tested as a beadlet blend alone and when tested as compressed tablets.

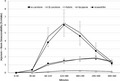

## 1 INTRODUCTION

Carotenoids are a major class of phytonutrients that provide much of the color found in fruits and vegetables. Of the 500+ naturally occurring carotenoids, only six have been well studied for their association with human health; β‐carotene, α‐carotene, lutein, zeaxanthin, lycopene, and astaxanthin. Epidemiological studies suggest that consumption of carotenoid‐rich foods is beneficial for human health. Because of this, health policies around the world recommend the consumption of fruits and vegetables at ≥400 g/day (5–9 servings) to encourage carotenoid intake in amounts sufficient to support better human health outcomes. However, most people find it difficult to consume enough carotenoid‐rich foods to consistently achieve recommended amounts (Murphy et al., 2014). One way to bridge dietary gaps of carotenoids is through supplementation (Marra & Bailey, 2018). Various forms and doses of carotenoid supplements exist and often, for convenience and simplicity, the primary six carotenoids of interest are consumed in mixtures, that is, multicarotenoids. However, bioavailability of carotenoids is complicated by many factors (Bohn, 2008). There exist numerous observations suggesting significant interaction and competition between various carotenoids during absorption and metabolism that can result in the inhibition of uptake of one carotenoid over the other. Specifically, mixed carotenoids, upon delivery into the intestinal system, compete for micelles and uptake into enterocytes for subsequent delivery into the lymph system and the bloodstream (Berg, 1999; Berg & van Vliet, 1998; Hof et al., 2000; Maiani et al., 2009).

Previously, we have tested multicarotenoid formulations utilizing specialized beadlets designed with the capability of separating individual carotenoid delivery one from another within the transit time of the digestive system. Gellenbeck et al., 2012 reported in vitro testing of a multilayered beadlet design that resulted in a 2–3 hr separation between lycopene, β‐, and α‐carotene peak release under simulated gastrointestinal conditions (Gellenbeck et al., 2012). In a separate clinical evaluation, a formulation designed to deliver a sequential release of a series of carotenoids was shown to limit interactivity one from another, resulting in improved carotenoid bioavailability in human subjects (Salter‐Venzon et al., 2017).

Here, we present the release profile of a blend of carotenoid beadlets designed to separate individual carotenoids one from one another (Table 2) when tested using a gut simulation model (TIM). The TIM is an in vitro model that provides a unique ability to sample and measure foods, formulations and compounds throughout the digestion process, providing insight into their predicted digestibility and bioaccessibility within a human intestinal tract.

## 2 MATERIALS AND METHODS

### 2.1 TIM model and test conditions

Experiments were conducted at The TIM Company (TIM B.V., The Netherlands). The dynamic, multi‐compartmental in vitro system of the stomach and small intestine (TIM‐1) has been described in detail in several publications (Bellmann et al., 2014; Domoto et al., 2013; Helbig et al., 2013; Minekus et al., ,^1995^, ^2005^; Van Loo‐Bouwman et al., 2014). Briefly, the TIM‐1 system consists of a stomach compartment and three small intestinal compartments, the duodenum, jejunum, and ileum. Each compartment is composed of two glass units with a flexible silicone inner wall enclosing the luminal material. The space between the inner and outer walls is filled with water. Peristaltic mixing of the chyme results from alternate compression and relaxation of the flexible inner wall. The compartments are connected by peristaltic valve pumps that successively open and close, allowing the chyme to transit over time through the compartments. In the stomach segment hydrochloric acid, α‐amylase, pepsin, and lipase are added while in the small intestinal compartments bicarbonate, electrolytes, pancreatic juice, and bile are added as described previously (Bellmann et al., 2016). The experimental test conditions including fluids, meal, pH, and timing are summarized in Table 1.

**TABLE 1:** Parameters simulated in the TIM‐1 systems describing the average gastrointestinal physiological conditions of healthy young adults after intake of a high‐fat meal (HFM)

TIM−1	Fed state (HFM)
Gastric compartment	
Intake (total)	300 g
Meal	150 g
Water and artificial saliva	140 g
Gastric start fluid	10 g
Gastric emptying T ½	80 min
Housekeeper wave	180 min
Gastric pH	6.5–1.7 over a 180 min period
Small intestinal compartments	
pH duodenum	5.9
pH jejunum	6.5
pH ileum	7.4
Experimental duration	6 hr

Prior to the performance of each experiment detailed here, the secretion fluids (e.g., gastric juice with enzymes, electrolytes, bile, and pancreatic juice) were freshly prepared, the pH electrodes calibrated, and semipermeable membrane (hollow fiber) units installed. All experiments were performed under yellow light with exclusion of daylight.

For simulation of the fed state conditions, a high‐fat standard meal was used as recommended by the U.S Food and Drug Administration (FDA) and the Center for Drug Evaluation and Research (CDER) for food‐effect bioavailability and fed bioequivalence studies (USDHHS_FDA_CDRE, 2002). This meal contains approximately 50% energy from fat, 20% energy from protein, and 30% energy in the form of carbohydrates. The meal is composed of eggs, bacon, toast bread, potatoes, milk, butter, and margarine. The meal was prepared as one batch, divided into portions of 150 g and stored at <−18°C. One portion of the meal was used for each TIM run.

For experiments with the tablet format, a sinker basket (Japanese_Pharmacopoeia, 2016) was used to allow the tablets to move together through the TIM system with full fluid contact. To mimic the housekeeper wave, the basket with the tablets was manually moved from the stomach compartment to the duodenum compartment where the tablets then disintegrated almost immediately.

### 2.2 Carotenoid beadlets and tablets

The active ingredient sources contained in the beadlets and tablets are as follows: Natural β‐carotene derived from *Dunaliella* algae and palm fruit, natural α‐carotene derived from palm fruit, lutein and zeaxanthin from marigold flowers, lycopene from tomato, and astaxanthin from *Haemotococcus* algae. These extracts were combined with standard inert ingredients by Omniactive Health Technologies (Morristown, NJ) using proprietary technology to form three of the carotenoid beadlets described in Table 2. The fourth beadlet containing astaxanthin was provided by Beijing Ginkgo Group (Irvine, CA).

**TABLE 2:** Carotenoid content in experimental Beadlets and Tablets

Beadlet	Carotenoids	Source	Release Profile Design	Amount in 270 mg Beadlet Blend Dosage (mg)^a^	Amount in 3 Tablet Blend Dosage (mg)^a^
1	Lutein	Marigold	Immediate after the stomach	5.7	6.5
	Zeaxanthin	Marigold	Immediate after the stomach	1.0	1.2
2	Astaxanthin	*Hematococcus* algae	Immediate after the stomach	0.266	0.275
3	Lycopene	Tomato	1–2 hr after the stomach	3.9	4.2
4	β‐carotene	*Dunaliella* algae	3–4 hr after the stomach	9.0	9.2
	α‐carotene	*Dunaliella* algae	3–4 hr after the stomach	1.5	1.6

Note:

All carotenoid beadlets were supplied by Omniactive Health Technologies, except astaxanthin which was supplied by Beijing Ginkgo Group.

^a^ Triskelion company assay.

Experiments were completed for both a blend of the free beadlets and the beadlets when compressed into tablets, which is a common delivery format. For the blend, four different beadlets were combined, providing 6 different dietary carotenoids in a specified ratio (Table 2). Beadlets containing lycopene, or β‐carotene and α‐carotene were formulated to delay peak release within the intestinal tract. For the tablet design, 270 mg of the beadlet blend was compressed into 3 tablets, in a background of crystalline cellulose, maltodextrin, lactose, croscarmellose sodium, magnesium stearate, and silicon dioxide, all ingredients that are standard carriers and excipients used for formulating tablets. Each tablet weighed an average of 480 mg and displayed a dissolution time of 16 min (US_Pharmacopoeia, 2017).

### 2.3 Sampling

During each TIM run, timed samples were taken at four points: jejunum filtrates, ileum filtrates, ileum effluent, and residue samples. The filtrate samples from the jejunum and ileum compartments were considered to contain the bioaccessible fractions while the ileum effluent and residue samples were considered nonbioaccessible in the upper gastrointestinal tract. All samples were stored at <−18°C until analysis.

### 2.4 Carotenoid analysis

Analysis of α‐carotene, β‐carotene, lutein, lycopene, and zeaxanthin was conducted at Triskelion (DUCARES B.V. trading as Triskelion). Postgut simulation samples were dissolved in tetrahydrofuran (THF) and saponified with THF‐ethanol/potassium hydroxide‐hexane overnight. The pH was adjusted to neutral and carotenoids were extracted with hexane. Hexane was evaporated and carotenoids were redissolved in acetonitrile/methanol/dichloromethane (ACN/MeOH/DCM). Analysis of lutein, zeaxanthin, lycopene, α‐carotene, and β‐carotene was performed using reversed‐phase chromatography with diode array detection, quantifying at 450 nm with DMT (rac‐5,7‐dimethyltocol) as the internal standard.

A different Triskelion method was used to quantify astaxanthin. Postgut simulation samples were dissolved in ethanol in the presence of the internal standard apo‐carotenal and cholesterol esterase solution was added. The samples were incubated at 37°C for 45 min. After cooling, sodium sulfate was added, and the solutions were extracted with petroleum ether. The petroleum ether was evaporated, and astaxanthin was redissolved in ethanol. Analysis of astaxanthin was performed using reversed‐phase chromatography with diode array detection, quantifying at 474 nm with apo‐carotenal as internal standard.

Additional lycopene analysis was performed on reserve samples at the Tufts University laboratory (Melendez‐Martinez et al., 2013). Briefly, samples were mixed well with ethanol and extracted twice with hexane and ether, 1:1, with the upper layer collected after centrifugation. The samples were then taken to dryness under nitrogen and red light. After reconstitution in ethanol: ether (2:1), 50 ml was injected into an HPLC system using a YMC C30 column for qualitative determination.

### 2.5 Data calculation

The raw data were used for calculation of the bioaccessible fraction of carotenoids for each time point during transit of the chyme through the TIM system.

The absolute amount of the carotenoids recovered in a sample was calculated by multiplying the analyzed concentration in the sample with the collected volume (Equation 1).

(1) $$A\;\left(\text{mg}\right)=C_{\text{sample}}\left(\mu g/ml\right)\times 10^{‐3}\times V_{\text{sample}}\left(\text{ml}\right)$$

The recovery of the carotenoids was determined by the sum of all amounts recovered in the filtrate fractions of the jejunum and the ileum, in the ileum effluent and in the residues and rinse fractions of the gastric, duodenum, jejunum, and ileum compartments. The total recovery is expressed as percentage of amount added to TIM (intake), as analyzed in the test product (Equation 2).

(2) $$\text{Recovery}\;\left(\%\right)=\frac{\Sigma A_{\text{filtrate}\;(\text{mg})}+\Sigma A_{\text{effluent}\;(\text{mg})}+\Sigma A_{\text{residues}\;(\text{mg})}}{A_{\text{intake}\;\text{(mg)}}}\times 100\%$$

The bioaccessibility was calculated by expressing the total amount of carotenoids recovered from the filtrate (jejunum plus ileum) as a percentage of the intake (Equation 3).

(3) $$\text{Bioaccessibility}\;\left(\%\;\text{of}\;\text{intake}\right)=\frac{\Sigma A_{\text{filtrate}\;(\text{mg})}}{A_{\text{intake}\;(\text{mg})}}\times 100\%$$

The results of the duplicate TIM‐1 runs in the main study were presented as mean ± SD (*n* = 2).

## 3 RESULTS

### 3.1 Bioaccessibility from the blend of free beadlets

**FIGURE 1:**
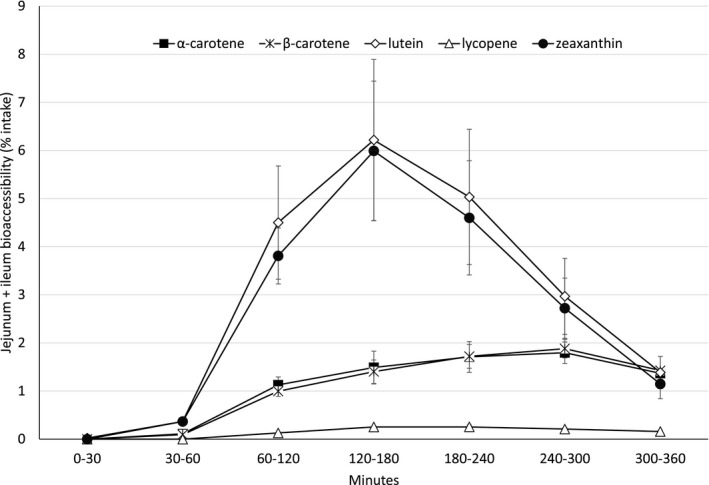
Time course of bioaccessibility profile of the free beadlet blend in the jejunum and ileum compartments combined

The bioaccessibility (% of intake) of carotenoids released from the blend of free beadlets in jejunum and ileum filtrate combined are shown in Figure 1 (Note: Results from the individual compartments can be found in the Appendix S1).

The maximal bioaccessibility of α‐ and β‐carotene was observed between 240 and 300 min. The accessibility profile shows a steady increase of release of these carotenes from the free beadlets until 300 min, at which point the accessibility decreases for the final hour of the experiment. However, the released carotenes did not approach zero at the termination of the run, suggesting that the total bioaccessibility of α‐ and β‐carotene was not complete by the conclusion of the experimental period (360 min).

The maximal bioaccessibility of lycopene released within the jejunal–ileal compartment was observed at an earlier time point than that seen with α‐ and β‐carotene, at between 120 and 180 min. Notably, the amount of lycopene was observed in very low levels, making distinction between the measurements difficult to discern.

The maximal bioaccessibility was observed between 120 and 180 min for lutein and zeaxanthin.

Absolute astaxanthin measurements were, in almost all cases, below the detection limit for the analysis (Limit of Quantification=300 μg/kg). Thus, they were not analyzed for bioaccessibility in this report.

### 3.2 Bioaccessibility from the beadlets compressed into tablets

**FIGURE 2:**
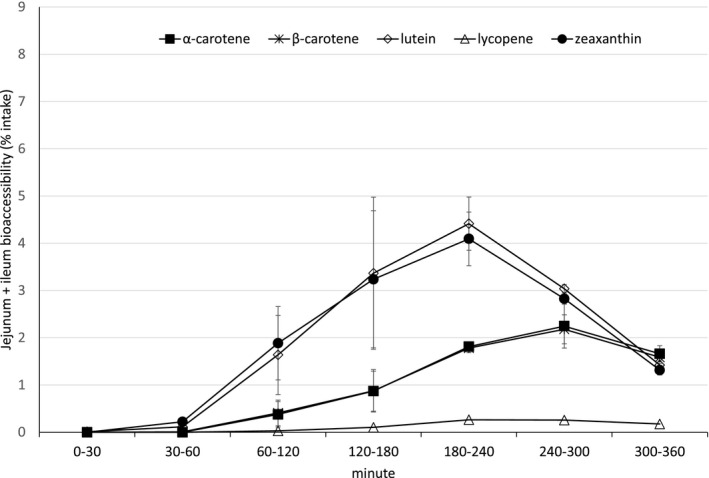
Time Course Release Bioaccessibility Profile of the Tablets in the jejunum and ileum compartments combined

The bioaccessibility (% of intake) of carotenoids from the tablet experiments in jejunum and ileum filtrate combined are shown in Figure 2 (Note: Results from the individual compartments can be found in the Appendix S1). Similar to the results seen in the experiments evaluating the bioaccessibility of the free beadlet blend, astaxanthin measurements from the compressed tablets were not sufficiently above the method detection limit and are therefore not considered here.

The maximal bioaccessibility of α‐ and β‐carotene obtained from jejunum and ileum filtrate was observed between 240 and 300 min. The bioaccessibility profile shows a steady increase of these carotenes until 300 min, at which point the bioaccessibility decreases toward the termination of the run. The profile suggests that α‐ and β‐carotene were not yet completely released from the compressed tablet by the end of the experimental period (360 min).

The maximal bioaccessibility of lycopene released within the jejunal–ileal compartment was observed at an earlier time point than that seen with α‐ and β‐carotene, at between 180 and 300 min. Again, as seen with the free carotenoid beadlets, the amount of lycopene was observed in low levels, making distinction between the measurements difficult to discern and the maximal plateau appears relatively flat.

In contrast, lutein and zeaxanthin showed a distinct peak in bioaccessibility between 180 and 240 min.

### 3.3 Cumulative bioaccessibility

**FIGURE 3:**
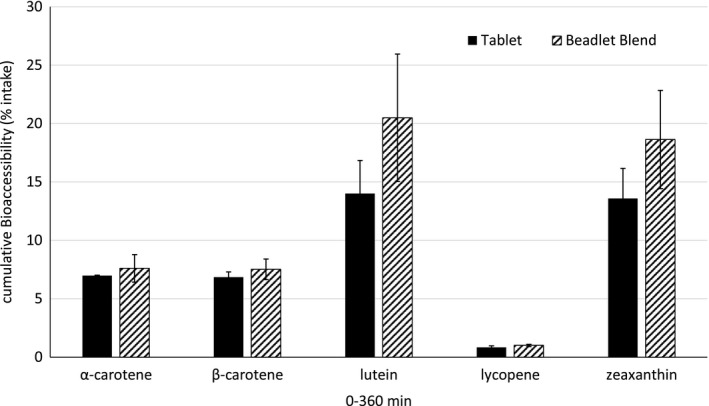
Carotenoid Total Bioaccessibility (% of Intake) for both the Beadlet Blend and Tablet Experiments

An overall comparison of cumulative carotenoid bioaccessibility (% intake) between the free beadlet blend and the compressed tablet is shown in Figure 3. The highest cumulative bioaccessibility was seen for lutein and zeaxanthin, with 20.5% of lutein and 18.6% of zeaxanthin recovered from the free beadlet blend, and 14.0% of lutein and 13.6% zeaxanthin recovered from the compressed tablet. Cumulative bioaccessibility for α‐carotene was 7.6% and 7.5% for β‐carotene recovered from the free beadlet blend, and 7.0% and 6.9% α‐ and β‐carotene, respectively, recovered from the compressed tablet. Lycopene bioaccessibility was significantly lower than the other carotenoids as only 1.0% and 0.8% of lycopene was recovered from the free beadlet blend or the compressed tablet, respectively.

### 3.4 Recovery of carotenoids

To calculate the total amount of each carotenoid recovered, the amount recovered from each bioaccessible filtrate (described above) was added to that recovered from the ileum effluent as well as the recovered amounts from each rinse fraction and residue from the gastric, duodenum, jejunum, and ileum compartments. The total values from both the Beadlet Blend and Tablet experiments are provided in Table 3.

**TABLE 3:** Average % Recovery for Beadlet Blend and Tablet Experiments

	Beadlet Blend	Tablet
α Carotene	69.0 ± 9.2	73.2 ± 7.9
β‐carotene	66.9 ± 11.4	76.8 ± 7.4
Lutein	84.5 ± 22.9	90.2 ± 6.8
Zeaxanthin	83.4 ± 21.6	86.7 ± 6.5
Lycopene	55.8 ± 1.0	53.3 ± 4.0

## 4 DISCUSSION

It is widely understood that consumption of carotenoid‐rich foods is beneficial for human health, yet most people are unable to meet the dietary recommendations through foods alone. For this reason, the addition of supplements containing carotenoids can be useful. As such, when multiple types of carotenoids are delivered into the gut, there can be interactions which inhibit bioavailability (Bohn, 2008). For this reason, specialized beadlets with the capability to separate the delivery of individual carotenoids one from another within the digestive system have been developed and tested (Gellenbeck et al., 2012; Salter‐Venzon et al., 2017).

Here, we report the bioaccessibility profiles resulting from a blend of carotenoid beadlets when tested in an in vitro system designed to mimic the upper gastrointestinal tract (TIM). In the TIM‐1 system, the tested beadlets, whether as a blend or compressed into a swallowable tablet, showed a bioaccessibility profile that matched the designed release profile (Table 2), with lutein and zeaxanthin released first, followed by lycopene, and α‐ and β‐carotene shortly thereafter. This order aligns with the design of the beadlets, which were designed in a manner to promote a faster release of lutein and zeaxanthin, followed by lycopene, and later by α‐ and β‐carotene. Similar results have been shown previously from in vitro and clinical evaluations of a layered beadlet of analogous design as the blends tested here (Gellenbeck et al., 2012; Salter‐Venzon et al., 2017).

The bioaccessibility release profile of the carotenoids contained in a free beadlet blend (Figure 1) demonstrated a similar curve as that seen when the beadlets were compressed into a tablet formulation (Figure 2). However, it is noted that the time of maximal bioaccessibility (*t*_max_) was different between the two formulations. The free beadlet formulation showed maximal bioaccessibility between 120 and 180 min for lutein and zeaxanthin, whereas for the same carotenoids, the compressed tablet showed maximal bioaccessibility later at 180–240 min. The shifted release dynamics seen with the tablet formulation may be attributed to the performance of the housekeeper wave in TIM‐1, which was performed manually at 180 min of the experiment. This housekeeper wave serves to transfer the tablet remainder from the stomach compartment to the first part of the small intestine, the duodenum, and likely delayed the time at which the compressed tablet demonstrated maximal bioaccessibility.

A wide variety of factors are known to affect the uptake and consequent bioavailability of carotenoids. These factors include uptake competition, carotenoid type, the matrix in which the carotenoid is located, particularly the fat and oil content (Nagao et al., 2013), personal genetics, and the technique used to quantify the bioaccessibility measurement (Chacón‐Ordóñez et al., 2019). Here, using the TIM dynamic gastrointestinal model, we found different cumulative total bioaccessibility (Figure 3) for specific carotenoids whether delivered from the free beadlet blend or from beadlets compressed into a tablet. Lycopene bioaccessibility was extremely low; however, these measurements may not represent the entire bioaccessible component, as only one form of the lycopene molecule was tested and quantified (discussed further below). α‐ and β‐carotene both demonstrated a cumulative bioaccessible fraction in the range of 7%–8%, while lutein and zeaxanthin measured between 13% and 20% cumulative bioaccessibility. These values generally agree with other reports of carotenoid bioaccessibility evaluated by various in vitro assessments. Estevez‐Santiago et al., (Estévez‐Santiago et al., 2016) reported bioaccessibility of α‐carotene at 0%–4.6%, and β‐carotene at 1%–9.1% from various fruits in a static digestion model, while Rodrigues et. al., measured lutein bioaccessibility from fruits using a variety of methods, with reports of bioaccessibility between 8% to 25% (Rodrigues et al., 2016). Within the matrix of dried algal biomass, Tudor et al. measured lutein bioaccessibility using an in vitro batch model with different bile extracts in the range of about 17%–20% for lutein from *Chlorella pyrenidosa*, and 24%–37% for zeaxanthin and about 20% for β‐carotene from *Arthrospira plantensis* (Tudor et al., 2021).

The high‐fat meal matrix used in these experiments provided primarily saturated fatty acids with a much lower amount of monounsaturated fatty acids. Various studies have pointed out that enhanced bioavailability of less polar carotenoids like β‐carotene is observed with a higher ratio of unsaturated:saturated fatty acids (Failla et al., 2014; Yao et al., 2021) but our experiments were not designed to address this variable and further experimentation would be needed to fully understand the effect in the TIM model. However, the higher bioaccessibility measurements for the more polar lutein and zeaxanthin seen here suggest it could be a factor affecting our results. Work by Zhou et al. demonstrated increased β‐carotene bioaccessibility when different carrier oils were utilized to form stable nanoemulsions, further illustrating the complexity of interactions between dietary fat and carotenoids to affect uptake (Zhou et al., 2018).

The total carotenoid recovery measurements shown in Table 3 illustrate a different release dynamic between the free beadlet blend recoveries and those measured from the compressed tablet formulation, with a small, but notable, higher recovery from the tablets for all carotenoids, except lycopene. The recovery of α‐carotene, β‐carotene, lutein, and zeaxanthin were 70%–90% from either formulation, but the tablet formulation illustrated ~5%–10% over that recovered from the free beadlet blend. Combining these results with the higher bioaccessibility seen with the beadlet blend over the tablet format, suggests carotenoid beadlet compression can hinder the release of the carotenoids within the intestine to a small degree.

Lycopene digestion and absorption is a complex topic (Arballo et al., 2021) and here we show recovery of lycopene was lowest for both formulations (~55% of intake). The low recovery resulted in difficulty determining a *t*_max_ and suggests that the carotenoid may have degraded/oxidized or otherwise changed form during the experiment, so that the assay used to measure the base molecule no longer recognizes this altered form. A high amount of lycopene loss has been reported previously using a dynamic gastrointestinal model, particularly in the jejunum and ileum phases (Berni et al., 2020). Various mechanisms have been hypothesized to explain this difference in lycopene recovery compared to other carotenoids. A previous study demonstrated that lycopene degradation occurred through oxidation and enzyme catalysis during in vitro digestion (Blanquet‐Diot et al., 2009). Dos Anjos Ferreira, et. al., found that 2‐ene‐5,8‐lycopenal‐furanoxide, lycopene‐5,6,5’,6’‐diepoxide, lycopene‐5,8‐furanoxide isomer, lycopene‐5,8‐epoxide isomer, and 3‐keto‐lycopene‐5’,8’‐furanoxide were among the lycopene oxidation products formed during metabolism (Anjos et al., 2003). Another hypothesis exists around components of the test meal interacting with the lycopene molecule. The test meal used in this experimentation included bacon, adding the possible presence of metmyoglobin, a potential oxidative agent. Kopec, *et*. *al.,* demonstrated metmyoglobin served as an oxidant and was able to degrade both lycopene and β‐carotene (Kopec et al., 2017). The analytical method originally used in this experiment was not designed to detect lycopene oxidation products, but rather only the parent lycopene. To further investigate the loss of lycopene, reserve samples were assayed at Tufts University (courtesy of Dr. Xiang‐Dong Wang). While some polar metabolites were detected in these assays, it was not possible to determine if they originated from lycopene or other carotenoids. Further experiments designed with lycopene alone and with carotenoid combinations, using detailed analytical methods will be needed to conclusively determine the fate of lycopene and its possible oxidation during simulated gastrointestinal conditions. An understanding of the fatty acids available in the TIM in vitro digestion process model could also provide further insight into the complexity of the lycopene modification and uptake.

## 5 CONCLUSIONS

This study has shown a unique beadlet blend formulation released carotenoids in the TIM dynamic gastrointestinal model with peak bioaccessibility separated over about 3–4 hr in the order of lutein and zeaxanthin first, followed by lycopene, then finally α‐ and β‐carotene; both as a free beadlet blend and after the beadlets have been compressed into tablets. Bioaccessibility measurements were slightly higher for the free beadlet blend than those measured from the tablet formulation. Carotenoid recoveries, with the exception of lycopene, ranged from 7% to 20%, and are measurements similar to those reported previously in literature. Recovery of lycopene was low and requires further experimentation to determine its fate within a simulated digestion model.

## AUTHOR CONTRIBUTIONS

**Chun Hu:** Conceptualization (equal); Data curation (lead); Formal analysis (equal); Funding acquisition (supporting); Investigation (equal); Methodology (equal); Project administration (equal); Resources (supporting); Software (lead); Validation (equal); Visualization (equal); Writing‐original draft (lead); Writing‐review & editing (equal). **Dawna Salter‐Venzon:** Conceptualization (equal); Data curation (supporting); Formal analysis (equal); Funding acquisition (supporting); Investigation (supporting); Methodology (equal); Project administration (supporting); Resources (equal); Writing‐original draft (equal); Writing‐review & editing (equal). **Katja Lange:** Data curation (equal); Formal analysis (equal); Investigation (lead); Methodology (equal); Project administration (equal); Resources (equal); Software (equal); Validation (equal); Writing‐review & editing (supporting). **Annet Maathuis:** Data curation (supporting); Formal analysis (equal); Methodology (supporting); Resources (supporting); Validation (equal); Writing‐review & editing (supporting). **Susann Bellmann:** Conceptualization (supporting); Formal analysis (equal); Investigation (supporting); Methodology (supporting); Validation (supporting); Writing‐review & editing (supporting). **Kevin W. Gellenbeck:** Conceptualization (lead); Data curation (supporting); Formal analysis (equal); Funding acquisition (lead); Investigation (equal); Methodology (equal); Project administration (lead); Resources (equal); Supervision (lead); Validation (supporting); Visualization (equal); Writing‐original draft (equal); Writing‐review & editing (lead).

## ETHICAL STATEMENT

C Hu, D Salter Venzon, and K Gellenbeck are employees of Access Business Group which provided the funding for this work.

## Supporting information

Fig S1‐2
