# Supplementary material for: Evaluation of the bioaccessibility of a carotenoid beadlet blend using an in vitro system mimicking the upper gastrointestinal tract
Source: Food Sci Nutr. 2021 May 4;9(6):3289–96. doi: 10.1002/fsn3.2295 (PMC8194940; doi:10.1002/fsn3.2295)
Supplement: Supplementary file 1 — Fig S1‐2 [file FSN3-9-3289-s001.docx]

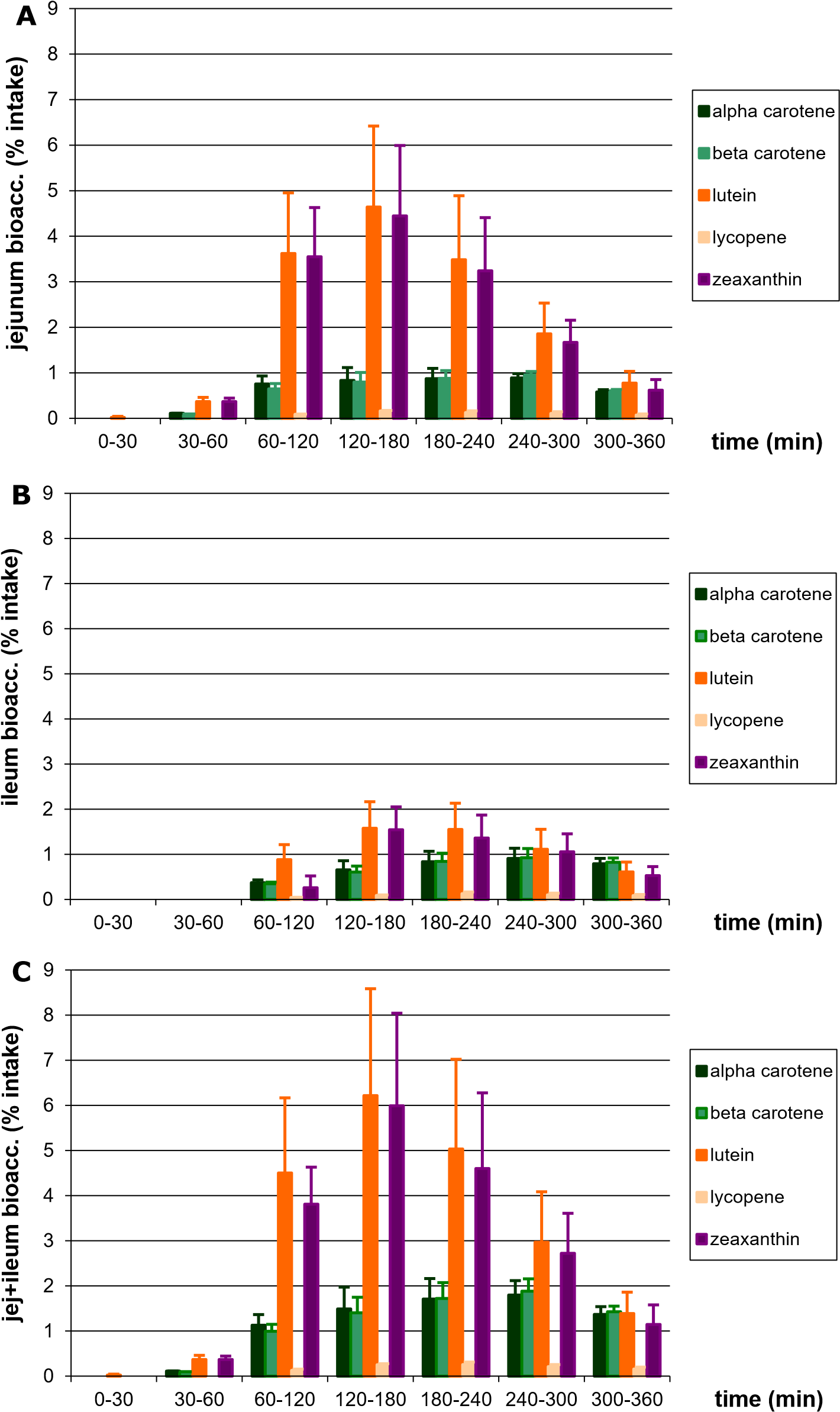


Bioaccessibility (as percentage of intake) from carotenoids in beadlet blend measured in filtrate from jejunum (A), ileum (B) and jejunum and ileum together (C) over time in 30 min and 60 min fractions (mean ± sd, n=2)


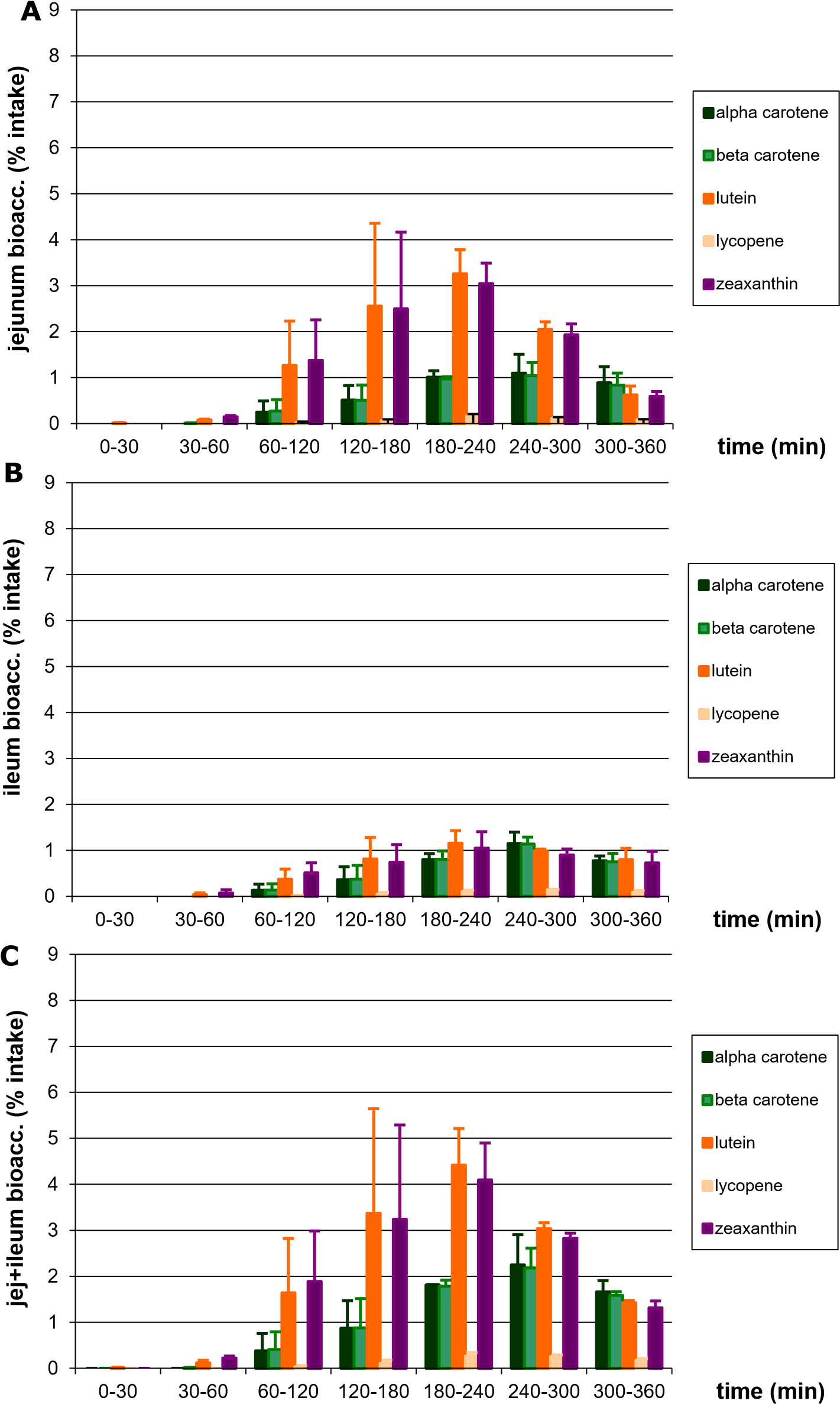


Bioaccessibility (as percentage of intake) from carotenoids in Tablet measured in filtrate from jejunum (A), ileum (B) and jejunum and ileum together (C) over time in 30 min and 60 min fractions (mean ± sd, n=2)
